# In situ surface dynamics in heterogeneous catalysis

**Published:** 2004-03-01

**Authors:** Kenzi Tamaru

**Affiliations:** Professor Emeritus, the University of Tokyo

**Keywords:** In situ studies, catalysis, dynamic relaxation, isotope jump method, working catalyst

## Abstract

During the former half of the last century the mechanism of heterogeneous catalysis had been studied, keeping the catalyst in a black box, and on the basis of the information outside of the black box, it was discussed just from mere conjectures. The author initiated a method to study directly the behavior of the working catalyst surface, looking into the inside of the black box by measuring adsorption on the working catalyst surface. In the same period many varieties of recent physical tools to study the solid surfaces have been developed and were applied to study the in situ dynamics of working catalyst surface. However, even if some chemisorbed species were observed on the working catalyst surface, it does not follow that they are reaction intermediates. A new dynamic approach to identify the dynamic behavior of each of the chemisorbed species under the reaction conditions, had been proposed by the author by use of “isotope jump method”, in which labeled species are replaced during the course of reaction to study the behavior of each of the adsorbed species under the reaction conditions. By using such new techniques we could not only identify the reaction path and the rate-determining step, but also new phenomena which are called “adsorption assisted processes” were discovered, It is, accordingly, hoped that by means of new nanotechnologies recently developed to study the behavior of single molecules on solid surfaces the nature of heterogeneous catalysis should make a remarkable advances on the basis of this in situ dynamic methods. In this review article emphasis has been put in the fundamental methods of dynamic approaches.

## Introduction

Surface catalysis has been studied very extensively in the past century. In the first half of the century, however, the catalyst was always kept in a black box, and only the entrance and exit of the black box have been examined during the course of the catalytic reaction. On the basis of those indirect information obtained from outside of the black box, such as, for instance, the kinetic behavior of the catalytic reaction, the mechanism of the reaction had been discussed from mere conjectures. In many cases the Langmuir-Hinshelwood or Eley-Rideal mechanism was used to interpret the kinetic behavior. Without studying the dynamic behavior of working catalyst surface, however, no real nature of catalysis could be elucidated. In this sense the field of heterogeneous catalysis was considered as an “art” rather than real science, although it was playing an important role in the applied field.

It was in 19581) (and later in the chapter of *Advances in Catalysis* in 19642)) when a new method to study in situ dynamic behavior of catalyst surface was first proposed by the author. He directly measured the adsorption on the working catalyst surface for the first time, looking into the real working catalyst in the black box. This new idea was rapidly and successfully developed by the aid of many varieties of new spectroscopic techniques such as IR and the electron spectroscopy, which appeared just after it. Under those circumstances the adsorbed species on the working catalyst could be examined in these periods. However, although some chemical species were being adsorbed on the working catalyst surface, it does not follow that they are real reaction intermediates through which the overall reaction proceeds. The author further developed a new technique, which is called “the isotope jump method”, to identify the real reaction intermediates and reaction path.3) This is the most orthodox in situ approach to elucidate the mechanism of heterogeneous catalysis, directly examining the dynamic behavior of each of the adsorbed species during the course of catalytic reaction. As the results of employing those new techniques a new phenomenon, “adsorption assisted processes” was discovered. In this manner the field of catalysis became a part of real scientific chemistry in the latter half of the last century.

## Decomposition of GeH_4_ on Ge surface

**Fig. 1:**
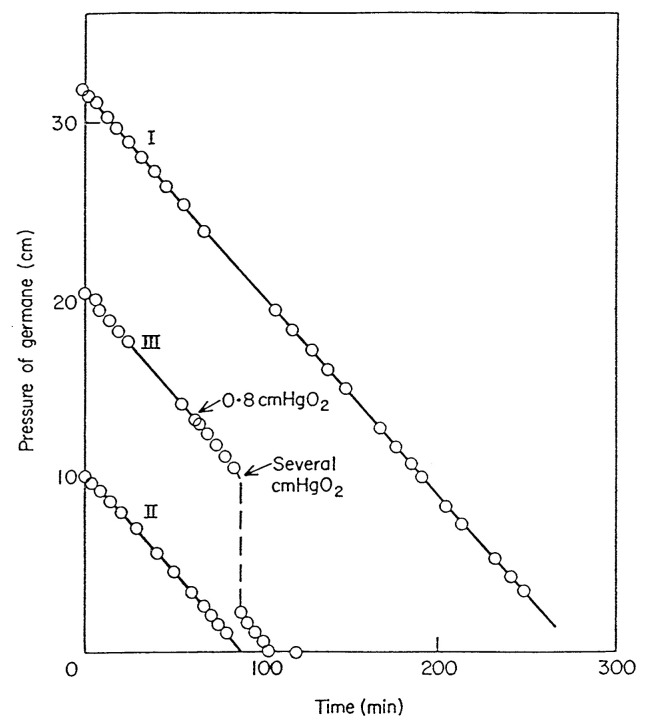
Decomposition of GeH_4_ on Ge surface at 551 K.

GeH_4_ = Ge + 2H_2_ The decomposition of GeH_4_ on Ge surface is one of the simplest catalytic reactions, having only two elements including catalyst. During the reaction the surface of the Ge catalyst is always renewed by the continuous deposition of fresh surface atoms. This reaction is (1) a beautiful zero order reaction, as given in Fig. 1, the rate being independent of the pressure of GeH_4_ and hydrogen; (2) the adsorption of hydrogen on Ge surface is reversible and dissociative and the initial activation energy for adsorption is 14.6 kcal/mol, and the heat of initial adsorption is 23.5 kcal/mol; (3) no HD is formed during the decomposition of GeH_4_ in the presence of ambient excess D_2_, although H_2_-D_2_ exchange reaction to form HD readily takes place after the decomposition; (4) during the decomposition the entire surface of Ge is virtually covered by chemisorbed hydrogen atoms, the number of which is approximately equal to that of surface Ge atoms. It was successfully estimated by using a large Ge surface area and by rapidly cooling the working catalyst and then temperature desorption technique; (5) the desorption rate of chemisorbed hydrogen on Ge surface extrapolated to full coverage is equal to the decomposition rate of GeH_4_.4),5)

The observations (3) and (4) demonstrate that the chemisorbed hydrogen during the reaction has much higher fugacity than the ambient hydrogen gas, in other words, the desorption of fully covered hydrogen is the rate-determining step of the overall reaction. In this way this catalytic system still remains as one of the cases of which reaction mechanism is well understood. In this manner this reaction system became the first example in the history of catalysis research of estimating adsorption on the working catalyst surface during catalytic reaction.

## Adsorption measurements on the working catalyst

The success of measuring adsorption during the course of catalytic decomposition of GeH_4_ suggested a new general method to study adsorption on the working catalysts which had never been carried out at that time. As the first catalytic reaction to study with this new general technique the decomposition of ammonia on tungsten surface was selected. This reaction is frequently discussed in the textbooks of catalysis as an example of zero-order reaction in its initial stage of the reaction, which is interpreted to indicate that the catalyst active surface is to be fully covered by ammonia molecules during the reaction.6) However, it was reported, on the other hand, that a rapid first step of the decomposition is the formation of hydrogen gas, which takes place at temperatures as low as 423 K and is followed by surface nitride formation at about 593 K.7) These results clearly indicate that ammonia molecules can not be the species that saturates the catalyst surface at the decomposition temperatures as high as above 873 K. Consequently, the species which saturates the active surface may be the chemisorbed nitrogen, of which desorption being the rate determining step. However, on the other hand, the kinetic isotope effect was detected to decompose NH_3_ more rapidly than ND_3_, which contradicts with the mechanism of nitrogen desorption to be the rate determining step.8) Under those circumstances, one of the direct methods to solve the mechanism of the reaction is to measure directly the adsorption during the reaction to identify the species which saturates the active part of the surface.

**Fig. 2:**
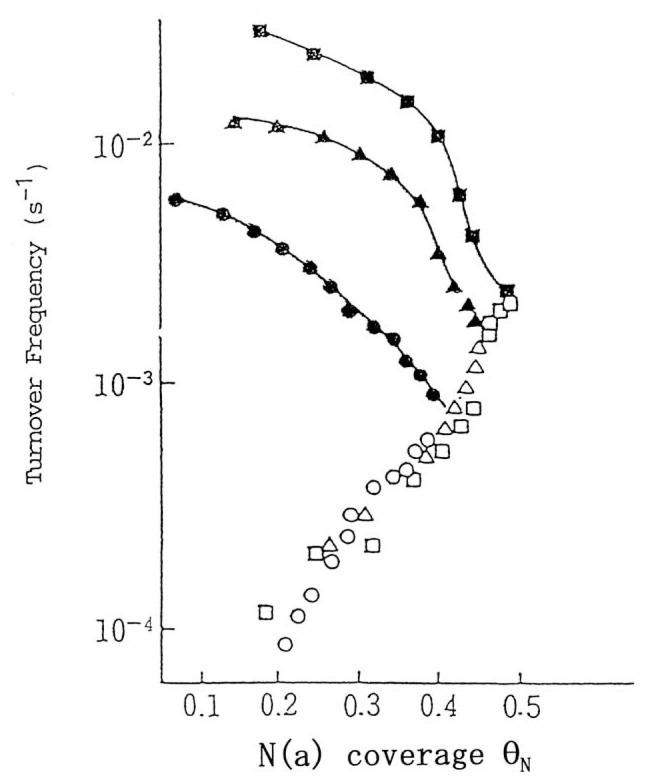
Rates of nitrogen uptake from ammonia and nitrogen desorption plotted against nitrogen coverage on tungsten at 1073 K. Ammonia pressure: ●, ○; 1.7 × 10^−6^Pa: ▲, △; 5.1 × 1^0–6^Pa: ■, □; 9.8 × 10^−6^Pa. ●, ▲, ■; V_in_ = −d(NH_3_)/n_W_dt: ○, △, □; V_out_ = 2dN_2_(g)/n_W_dt.

When ammonia is introduced onto a clean tungsten surface, hydrogen evolved and chemisorption of nitrogen takes place, which is rapid at first and subsequently slow down as more nitrogen is chemisorbed. On the other hand, nitrogen desorption is slow at the beginning and then becomes more rapid, finally reaching its steady state. During such processes no appreciable amount of hydrogen is chemisorbed in any form at the reaction temperatures.9) The results are given in Fig. 2, where the rate (r_a_) of nitrogen uptake from ammonia onto the surface and the rate (r_d_) of nitrogen desorption from the surface to form nitrogen gas are given as the number of nitrogen atoms per second per surface tungsten atom.10) When both rates of r_a_ and r_d_ are equal, it becomes a steady state of the reaction. Under higher pressure of ammonia more than full coverage of nitrogen is chemisorbed which demonstrates surface nitride layer formation, and the thicker the nitride layer becomes, the slower the nitrogen uptake, whereas the faster the nitrogen gas formation,9) which is similar to Fig. 2. The rate of the overall decomposition reaction is first order with respect to ammonia at lower ammonia pressure and approaches zero order at higher ammonia pressures. The rate is always independent of hydrogen pressure. The kinetic isotope effect of hydrogen, accordingly, is involved in r_a_, which gives higher coverage of nitrogen for NH_3_ than ND_3_ although no hydrogen chemisorbed is present in any form on the working surface.

When the adsorption isotherm of nitrogen on tungsten is studied, it is readily concluded that the nitrogen chemisorbed during the course of reaction is markedly more than that in adsorption equilibrium with ambient nitrogen gas. There is a chemical potential drop between the ambient nitrogen gas and chemisorbed nitrogen. The fact that hydrogen does not affect the rate of nitrogen desorption, strongly suggests that no equilibrium among ambient ammonia gas, hydrogen gas and chemisorbed nitrogen is realized. In this manner it was demonstrated for the first time by the in situ dynamic studies of the working catalyst surface that the ammonia decomposition on tungsten is to be interpreted by the dynamic balance mechanism between the supply and desorption of chemisorbed nitrogen, which is markedly different mechanism from what had been generally accepted.

The rate of ammonia decomposition on many transition metals, on the other hand, is expressed by the following empirical equation:

[1] $$\text{Rate of reaction}=\text{k}{\left({{\text{P}}_{\text{A}}}^{2}/{{\text{P}}_{\text{H}}}^{3}\right)}^{\text{n}}$$

where P_A_ and P_H_ are the pressures of ammonia and hydrogen, respectively, and k and n are constants. This kinetic equation is very interesting in the sense that the rate is strongly retarded by hydrogen, which is one of the reaction products. According to the Temkin-Pyzhev mechanism,11) the equation [1] is well explained by assuming that nitrogen desorption is the only rate-determining step and that a quasi-equilibrium among gaseous ammonia, chemisorbed nitrogen and gaseous hydrogen exists in the steady state of the reaction:

[2] $${\text{NH}}_{3}(\text{g})=\text{N}(\text{a})+(3/2){\text{H}}_{2}(\text{g})$$

According to this mechanism, in the steady state of the reaction the nitrogen chemisorbed on the catalyst surface, N(a), is not in adsorption equilibrium with the ambient nitrogen gas, but with the “virtual pressure” (P_N_^*^) of nitrogen which is in equilibrium with the ambient ammonia and hydrogen as follows: P_A_^2^/(P_N_^*^P_H_^3^) = K_e_, where K_e_ is the equilibrium constant of the reaction; N_2_(g) + 3H_2_(g) = 2NH_3_(g). The mechanism, proposed by Temkin and Pyzhev,11) has been supported by various experimental observations, as is explained in various review articles.

It is interesting to note that on many transition metal catalysts ammonia decomposes according to the equation [1], hydrogen markedly retarding the reaction. However, on tungsten surface, on the other hand, the rate is only dependent upon ammonia pressure. In the case of platinum and iron the kinetics of the NH_3_ decomposition proceeds via the tungsten-type mechanism at higher temperatures and lower hydrogen pressures, whereas at lower temperatures and higher hydrogen pressures the decomposition proceeds according to the equation [1]. It was beautifully demonstrated that over the ruthenium (1,1,10) surface, the former mechanism is operative on its terrace sites, whereas the latter mechanism is operative on the step sites under similar reaction conditions, where the in situ nitrogen coverage at the step sites and terrace, and also the nitrogen desorption rate therefrom were directly measured. 10) It is a very interesting problem to study the reason why such different behavior appears under different reaction conditions.

When ammonia decomposes on the catalyst surface, the rate of nitrogen chemisorption from ammonia (r_a_^A^) in the steady state of the reaction should balance with its rate of consumption. The latter is composed of two contributions; the rate of desorption of the chemisorbed nitrogen to form nitrogen molecules (r_d_^N^), and the rate of hydrogenation of the chemisorbed nitrogen to re-form ammonia (r_d_^A^). The supply of chemisorbed nitrogen from ambient nitrogen gas is generally negligible, since the chemical potential of chemisorbed nitrogen on the working surface is generally much higher than the ambient nitrogen gas in the steady state of the reaction.

[3] $$2{{\text{r}}_{\text{a}}}^{\text{A}}=2{{\text{r}}_{\text{d}}}^{\text{A}}+{{\text{r}}_{\text{d}}}^{\text{N}}$$

If the nitrogen chemisorption from ammonia mostly goes back to ammonia, reacting with hydrogen, then 2r_d_^A^ is much larger than r_d_^N^. Consequently, the ratio r_a_^A^/r_d_^A^ approaches unity, which results in a quasi-equilibrium which is represented by equation [2]. The free energy drop accompanied by the step, accordingly, approaches zero, which is really the case in the Temkin-Pyzhev mechanism.

On the other hand, if 2r_d_^A^ is much smaller than r_d_^N^, most of the nitrogen that is supplied from ammonia will go to dinitrogen molecules to be desorbed before it is hydrogenated back to ammonia. This is the case in the tungsten-type mechanism, and there is an appreciable chemical potential drop in the step involving the supply of chemisorbed nitrogen from ammonia, the forward step of the equation [2]. The rate of the overall reaction is only dependent upon ammonia pressure and its order of the reaction is between first and zero order as to ammonia pressure.

On the basis of such discussion it is clear that the ratio, r_d_^N^/2r_d_^A^, which is expressed by *γ*, is a parameter that determines which of the two mechanisms is operative. If it is much larger than unity, then the tungstentype mechanism should be operative, whereas if it is much smaller than unity, then the Temkin-Pyzhev mechanism should be observed. This criterion certainly applies to ammonia decomposition and its synthesis. Since r_d_^N^ has the activation energy for the desorption of chemisorbed nitrogen, which is higher than that for its hydrogenation in r_d_^A^, *γ* will become larger at higher temperatures, while at higher hydrogen pressures, *γ* becomes smaller, since the hydrogenation of chemisorbed nitrogen proceeds faster. In this manner the behavior of ammonia decomposition and synthesis has been reasonably explained on the basis of such general mechanism,12) and is beautifully demonstrated in the case of platinum catalyst as given in Fig. 3.13)

The dynamic relaxation method: isotope jump method: The chemical potential of reaction intermediates in the steady state of reaction: When a catalytic reaction, A = B, would proceeds via several reaction intermediates, I_1_, I_2_, ···, I_n−1_, as follows:

[4] $$\text{A}⇋{\text{I}}_{1}⇋{\text{I}}_{2}⇋\cdots ⇋{\text{I}}_{\text{n}-1}⇋\text{B}$$

at each elementary step (i) the rates of forward and backward steps, v_+I_, and v_−I_, respectively, their difference v_i_,(v_+I_ − v_−I_), and the free energy change accompanied by the step, ΔG_i_, may be correlated as follows:

[5] $${\text{v}}_{+\text{i}}/{\text{v}}_{-\text{I}}=\text{exp}(-\Delta {\text{G}}_{\text{i}}/\text{RT})\;\text{or }{\text{v}}_{\text{i}}={\text{v}}_{+\text{I}}(1-\text{exp}(\Delta {\text{G}}_{\text{i}}/\text{RT}))$$

where R and T are gas constant and temperature, respectively, and −ΔG_i_ may be called “affinity” of the step. In the steady state of the reaction the rate of the overall reaction, V, may be expressed as follows:

[6] $$\text{V}=(1/{\text{s}}_{1})({\text{v}}_{+1}-{\text{v}}_{-1})=(1/{\text{s}}_{2})({\text{v}}_{+2}-{\text{v}}_{-2})=\;\cdot \;\cdot \;=(1/{\text{s}}_{\text{n}})({\text{v}}_{+\text{n}}-{\text{v}}_{-\text{n}})$$

where s_i_ is the stoichiometric number of each of the steps, which means the number of times which the step i should be repeated for the overall reaction to take place once. On the other hand, the free energy change accompanied by the overall reaction, ΔG, may be expressed as follows:

[7] $$\Delta \text{G}=\Sigma {\text{s}}_{\text{i}}\Delta {\text{G}}_{\text{i}}$$

If the r-th step has much larger value of s_e_/v_+r_ than any of the other steps, then −ΔG_r_ becomes much larger than any of the −ΔG_i_ of other steps, and ΔG would become equal to s_r_ΔG_r_, accordingly,

[8] $$\text{V}=({\text{v}}_{+\text{r}}/{\text{s}}_{\text{r}})(1-\text{exp}(\Delta \text{G}/{\text{s}}_{\text{r}}\text{RT}))$$

**Fig. 4:**
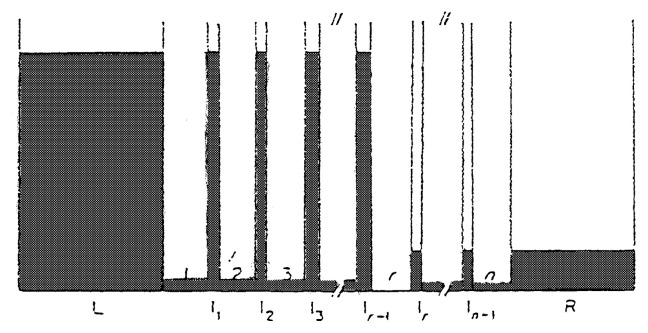
A series of water tanks connected by tubes of various sizes. (The r-th tube is the narrowest tube).

If all the s in the overall reaction is unity, such situation is schematically demonstrated in Fig. 4 for the system, where two big water tanks, A and B, are connected through many small tanks and the tube which connects between I_r−1_ and I_r_ is much narrower than any of the other tubes, then the water levels of each of the tanks will become something like Fig. 4. The capacity of the water tank, water level and the size of the tubes between the tanks correspond to the resident time in the tank, chemical potential and the rate constant of the step, respectively. The step where the major drop of the water level takes place (r-th step) may be called “rate-determining step”. When the overall free energy change, ΔG, is concentrated in the r-th step, the ratio of v_+i_/v_−i_ for all the other steps approaches unity and practically those steps are in quasi-equilibrium in the steady state of the reaction, The chemical potential of the reactant of the r-th step, consequently, becomes equal to that of the reactant, A, and that of the product of the r-th step becomes equal to that of the reaction product, B. In other words, V_+_/V_−_= v_+r_/v_−r_ = exp(−ΔG/s_r_RT).

It is evident, accordingly, the chemical potential of each of the reaction intermediates under reaction conditions is markedly dependent upon the location of the rate determining step. In the steady state of the overall reaction if the chemical potential of some of the intermediate species would be estimated by measuring the amount of adsorbed species, the level of the water tank may be estimated, which strongly suggests the location of the rate determining step. The cases of GeH_4_ and ammonia decomposition clearly indicate such situation.

If some dye (isotope) would be added to one of the water tanks (replacement to labeled species) in the steady state of the water flow or the reaction, the dye will go to the neighboring water tank at a rate depending upon the size of the connecting tube in the flowing (reacting) system. In this manner the rate of each of the steps may be estimated under the reaction conditions. The behavior of the dye may also tell the order of the sequence of reaction steps or the reaction path. During the course of catalytic reaction on the catalyst surface, even if some adsorbed species may be detected, it may just be staying on the catalyst surface, being independent of the reaction path. However, by using such dynamic “isotope jump method”, the sequence of the steps through which the overall reaction takes place may be identified. These techniques to give dynamic perturbations to study the nature of the flow are called “dynamic relaxation methods” by which we may estimate the in situ rate or rate constant of each of the elementary steps of the overall reaction. This new approach may be called “isotopic transient IR studies”,14) and other names.

Since the surface which behaves as catalyst is that in the working state where some adsorbed species are present in most cases depending upon the kinetic structure of the overall reaction, it is naturally very important to examine the catalyst surface while the reaction is taking place and also the behavior of each of the adsorbed species on such working catalyst surface.

## Adsorption measurements during the course of reaction and adsorption assisted processes

The adsorption on the catalyst surface while the reaction is taking place may be volumetrically measured using a closed circulation system. Such classical volumetric approach had been particularly effective in the case where only one kind of adsorbed species is present such as in the case of GeH_4_ and ammonia decomposition. Generally speaking, various kinds of gases would be adsorbed during the course of catalysis and the adsorption may be observed by means of many varieties of spectroscopic techniques. However, such volumetric measurements are advisable to measure along with the spectroscopic techniques, since some chemisorbed species are not easily quantitatively detected by the spectroscopic techniques.

In the spectroscopic measurements of adsorption on the working catalyst surface the reaction may be studied by flow systems and the adsorption on the working catalyst may be estimated by flash desorption technique and temperature programmed desorption. In many cases ultrahigh vacuum systems may be used to study catalysis on well defined catalyst surfaces. In recent years the techniques to study the behavior of each of the molecules on the well defined surface had remarkably advanced, which will certainly give much deeper insight into the real nature of heterogeneous catalysis.

The spectroscopic techniques to observe the adsorbed species on the working catalyst surfaces are certainly powerful technique to detect reaction intermediates. However, as mentioned before, even if some adsorbed species are detected, it does not follow that they are really reaction intermediates. In particular, the reactive species in the reaction sequences do not stay stably on the catalyst surface, whereas in many cases stable reaction byproducts stay long on the catalyst surfaces. Under those circumstances, in some cases we may detect only stable reaction byproducts on the catalyst surface. For example, in the hydrogenation of ethene on ZrO_2_ the dissociatively chemisorbed hydrogen which can be observed by IR is not the reaction intermediate.15) Accordingly, in elucidating the real reaction mechanism we have to study the dynamic behavior of each of the adsorbed species under the reaction conditions. This is the reason why we have to employ what we call “isotope jump method”.

**Fig. 5:**
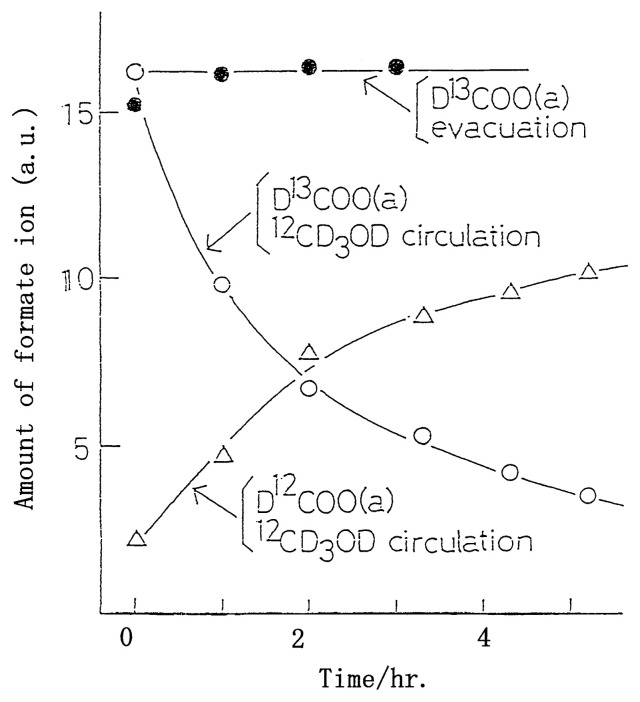
Behavior of chemisorbed surface formate on Cr_2_O_3_ during the course of methanol decomposition in the absence and presence of methanol at 533 K.

Let us take an example of this technique. During the course of methyl alcohol decomposition on Cr_2_O_3_, the formate ion could be detected on the working catalyst surface by means of infrared absorption technique.3) It is interesting to note that the formate ion on the surface is stable under vacuum at the reaction temperatures, which strongly suggests that the formate can not be the reaction intermediate through which the overall reaction proceeds. In the presence of methyl alcohol in the ambient gas, however, this formate ion behaves as a reaction intermediate, which was demonstrated by “the isotope jump method” as given in Fig. 5. In this figure it is clearly shown that the adsorbed formate ion, D^13^COO(a), stays very stable under vacuum at the reaction temperature, whereas in the presence of methyl alcohol, ^12^CD_3_OD, in the ambient gas, D^13^COO(a) was readily replaced by D^12^COO(a) and appears in the reaction products at a corresponding rate. In this manner the formate ion on the catalyst surface is the real reaction intermediate, although it is stable under vacuum at the reaction temperature. The reactivity of the surface formate ion is markedly increased by the presence of methyl alcohol, which may be called “adsorption assisted process.”

A similar behavior of the adsorbed species was reported in the case of ethyl alcohol dehydrogenation decomposition on a Nb monomer attached on SiO_2_ catalyst. 16) During the course of the reaction, although appreciable amount of adsorbed species are present on the catalyst surface, when all the vapor and gases are evacuated, the reaction stopped completely, the adsorbed species staying stably under vacuum. It is only at much higher temperatures where the decomposition of the adsorbed species takes place to dehydration decomposition. However, in the presence of alcohol in the ambient gas, then the adsorbed species reacted to proceed to dehydrogenation. In other words, the chemisorbed ethoxide decomposed being assisted by the presence of ethyl alcohol to dehydrogenation. The compounds with higher donor number at the nucleophilic level such as (C_2_H_5_)_3_N also assisted the decomposition of the ethoxide. In addition to such cases the decomposition of formic acid on Ni-SiO_2_ was also demonstrated that the reaction intermediate, formate ion on the catalyst surface, reacts more rapidly in the presence of formic acid in the ambient gas than under vacuum. 17)

The water gas shift reaction on various catalysts such as, for instance, MgO and ZnO, is another example of such adsorption-assisted reaction. In these cases the behavior of surface formate intermediates is markedly influenced by weakly coadsorbed water molecules.18) In some cases the introduction of CO causes catalytic reactions such as the decomposition of NH_3_ on Ru(001).19)

In this manner it is very interesting to find that the reactivity of some chemisorbed species is markedly increased by the presence of some species and spectators in the ambient gas, and some times the selectivity of the reaction can also be changed by the adsorption assisted processes. Accordingly, the temperature programmed reaction under vacuum to study the reactivity of chemisorbed species by raising the temperature under vacuum can not tell such adsorption-assisted processes, sometimes misleading the reaction mechanism. It is thus clearly demonstrated that the mechanism of catalytic reaction should be always studied under the reaction conditions.20)

According to the Langmuir adsorption-desorption kinetics, it is always assumed that the rate of adsorption is a function of pressure and the vacant surface available for adsorption, whereas that of desorption is the only function of the amount of adsorbed species, being independent of the presence of ambient gas. It is always described in this manner in all the textbooks in this field. Since more and more examples of “adsorption assisted processes” are being detected it was almost the time to examine the behavior of chemisorbed species on the basis of such view-points of adsorption assisted processes.

**Fig. 6:**
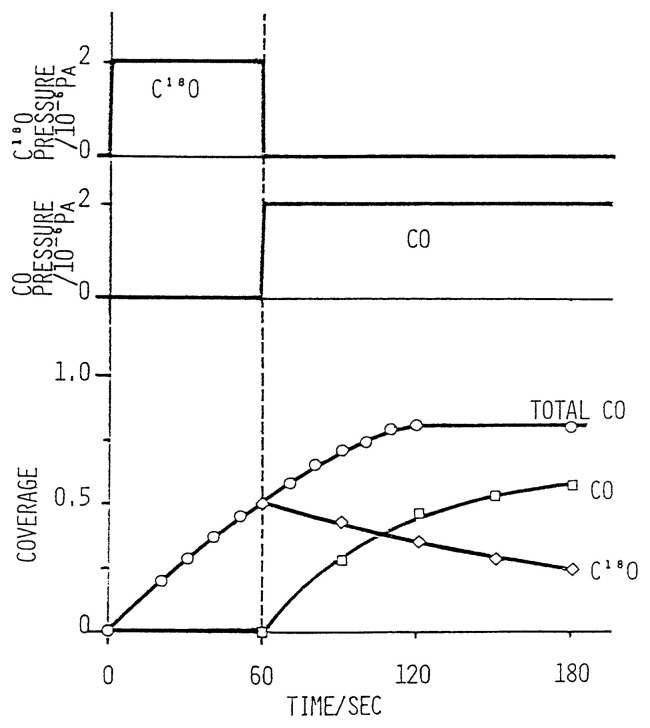
Isotope jump experiments during CO adsorption.

**Fig. 7:**
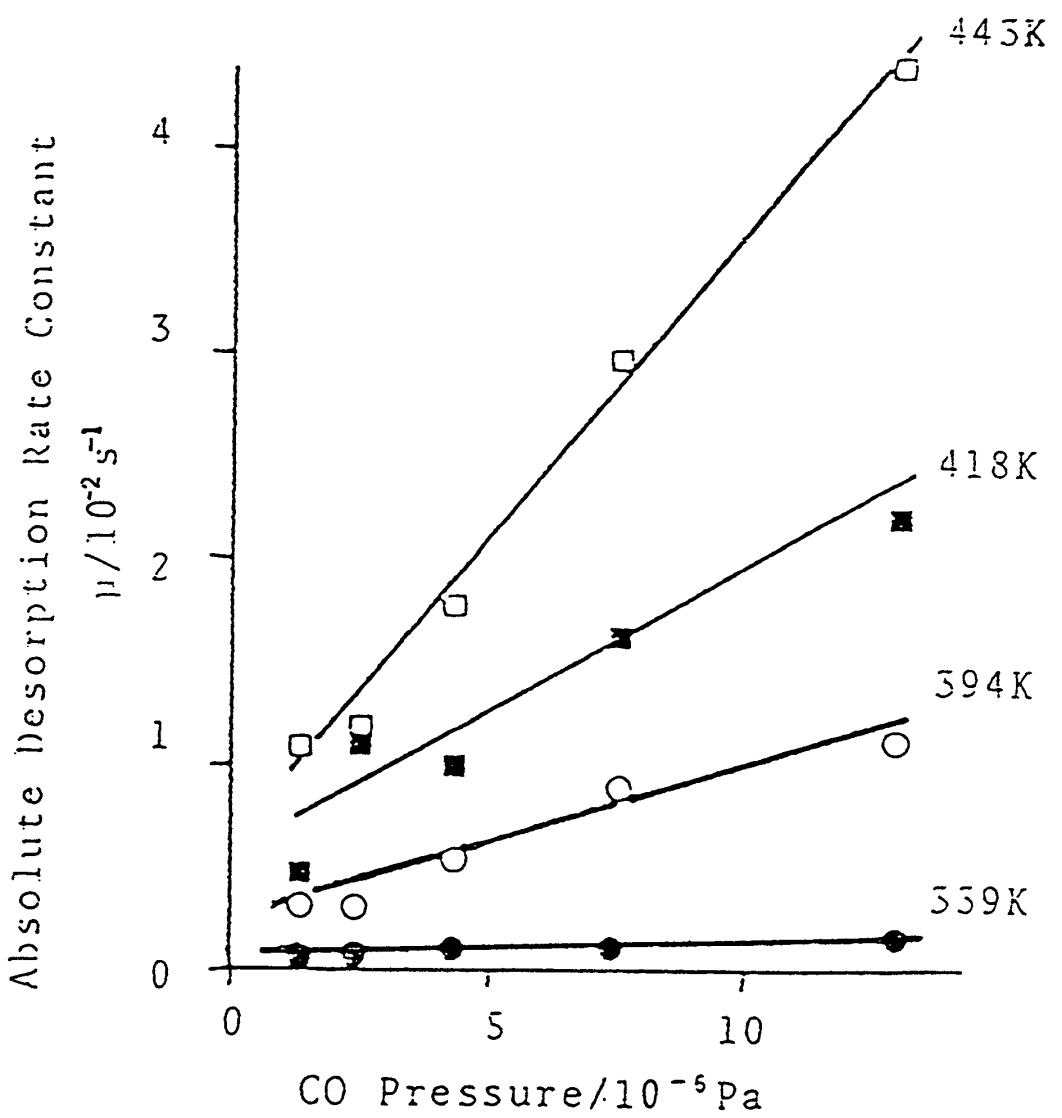
Dependence of the absolute desorption rate constant as a function of pressure and temperature: CO/Rh.

The isotope jump method was applied to study the kinetics of adsorption-desorption of CO on transition metal surfaces.21) During the course of adsorption of labeled *CO the ambient *CO was quickly replaced by non-labeled CO and the absolute rates of adsorption and desorption were measured from the subsequent rates of desorption of *CO and adsorption of CO at the coverage under the ambient CO pressure as given in Fig. 6. It is accordingly demonstrated that the absolute rate of desorption (r_d_) is markedly dependent upon the ambient pressure of CO gas as given in Fig. 7. In the case of Rh surface, the rate of adsorption (r_a_), on the other hand, is given as a typical Kisliuk mechanism,22) getting to zero at full coverage as given by the following equations.

[9] $${\text{r}}_{\text{a}}={\text{k}}_{\text{a}}\text{P}/[1+\text{K}\theta /(1-\theta )]$$

[10] $${\text{r}}_{\text{a}}={\text{K}}_{\text{D}}\theta (1+{\text{BP}}^{\text{N}})$$

where K and n are constants, k_a_ and k_d_, the rate constants, and *θ*, the coverage.21) The BP^n^ term in the equation corresponds to the enhancement of the desorption rate due to the incident molecules, which is called adsorption-assisted desorption. From the equations [9] and [10] the adsorption isotherm, equation [11], may be obtained.

[11] $$\theta \;[1+\theta \text{K}/(1-\theta )]=({\text{k}}_{\text{a}}/{\text{k}}_{\text{d}})\text{P}/(1+{\text{BP}}^{\text{n}})$$

According to the equation [11], which takes the adsorption-assisted desorption into account, when *θ* is as low as less than 1/3, *θ* increases more rapidly than the Langmuir adsorption isotherm as the pressure becomes higher. According to the adsorption isotherm the desorption is accelerated by the presence of ambient gas, in particular under higher pressures and coverages, and the apparent saturation appears at the coverages which depends upon the temperature. In this point this new adsorption isotherm is different from the Langmuir adsorption isotherm which gives a constant saturation values at different temperatures. The characteristic features of this new adsorption isotherm are in agreement with the adsorption isotherm experimentally obtained.21)

The interaction of preadsorbed CO with an incident CO molecule was studied on a Ni(100) surface by means of time-resolved infrared reflection absorption spectroscopy and the dynamic equilibrium between the readsorbed ^13^C^18^O and the gas ^12^C^16^O was directly examined. It was thus demonstrated that the labeled CO desorption process in the presence of non-labeled CO gas is markedly enhanced due to the repulsive interaction between the preadsorbed labeled CO and incident non-labeled CO molecules from the gas phase, arriving at nearby sites of preadsorbed CO. The activation energy is reduced from 127 kJ/mol, the value for desorption into vacuum, to 25 kJ/mol even for *θ* = 0.1~0.3 monolayer (ML) and a CO flux of 0.001 ML/s. Similar results were obtained on the Pt(111) surface.23)

It is very interesting to note that the isotope jump method demonstrated for the first time the adsorption-assisted process in the rate of desorption, although there are some experimental results in the literature which suggests it appears.24) The systematic study of the dynamics of adsorption-assisted processes, accordingly, successfully introduced a new concept into surface dynamics. The desorption is a process to break a bond between substrate and adsorbed species, but if the bond to be broken is the one in the adsorbed species, it can be an “adsorption-assisted catalytic reaction.”

## Recent advances in surface science and heterogeneous catalysis

In recent years the techniques to study the behavior of each of the single molecules chemisorbed on solid surface have been remarkably advanced. The vibrational spectra as well as dynamic behavior of single molecules, such as dissociation and hopping, can be studied. It is extremely interesting to apply such techniques to elucidate the real nature of the chemical reactions on solid surfaces. As is well known solid surface is very heterogeneous containing various different sites such as different crystal places, steps, kinks and defects. The electronic properties of these different sites depend upon the nature of each different sites, which results in different characteristic catalytic activity. It is very interesting to demonstrate the catalytic activity of each of the different sites on the catalyst surface.

Since it is becoming possible to observe the behavior of each of the chemisorbed species, the next step should be to examine the reactivity of each molecule chemisorbed on different sites to identify the real “active centers”. The isotope jump method should also be available for such purpose. However, in general, under the conditions to proceed chemical reaction on the surface, the chemisorbed species are easily mobile on the surface. Such mobility of the chemisorbed species should be avoided to identify the activity of reaction sites on the catalyst surfaces. One of the ways to overcome such difficulties would be to find out any reactions which takes place under the conditions no mobility takes place, which is in general not easy. Another possibility would be to study surface reaction under the condition such that the mobility is strictly limited, such as, for instance, the reaction at full coverage for the species which chemisorbs very strong. For instance, the reactants are strongly chemisorbed on the surface to have full coverage and the reaction such as isotope exchange reaction may be studied to detect the location of labeled species in the full coverage by means of nano-technological techniques. In such a manner, for instance, such recent advances in studying the behavior of each of the chemisorbed molecules may be applied to identify the real catalytically active sites on the surface. It is the time for us to be able to elucidate the catalytic reaction in molecular level on the catalyst surface, which had been a dream for a long time in the past.

## Figures and Tables

**Fig. 3:**
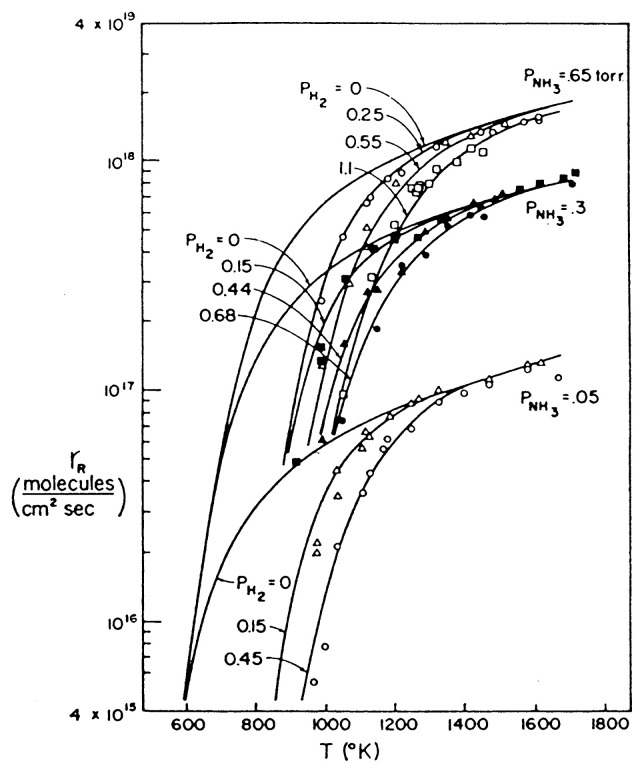
Plot of rate of ammonia decomposition versus T in NH_3_-H_2_ mixtures for three pressures of NH_3_ with variable H_2_ pressures as indicated. (Loeffler, D. G., and Schmidt, L. D. (1976) J. Catal. **41**, 440: Fig. 7).
